# Efficacy of hyperbaric oxygen therapy as an adjunct therapy in the treatment of sleep disorders among patients with Parkinson’s disease: a meta-analysis

**DOI:** 10.3389/fneur.2024.1328911

**Published:** 2024-07-31

**Authors:** Wei-qiang Tan, Qing Liu, Ming-jun Cen, Ian I. Leong, Zhao-quan Pan, Mu-xi Liao, Li-xing Zhuang

**Affiliations:** ^1^Guangxi University of Chinese Medicine, Nanning, China; ^2^Guangzhou University of Chinese Medicine, Guangzhou, China; ^3^Shenzhen Nanshan District Chinese Medicine Hospital (The First Affiliated Hospital of Guangzhou University of Chinese Medicine at Nanshan District, Shenzhen city), Shenzhen, China; ^4^The First Affiliated Hospital of Guangzhou University of Chinese Medicine, Guangzhou, China

**Keywords:** hyperbaric oxygen therapy, Parkinson’s disease, sleep disorders, efficacy, meta-analysis

## Abstract

**Objective:**

To systematically evaluate the efficacy of hyperbaric oxygen therapy (HBOT) as an adjunct therapy for treating sleep disorders in patients with Parkinson’s disease (PD).

**Methods:**

We conducted comprehensive searches in eight databases from inception through September 2023, including PubMed, Cochrane Library, Embase, Web of Science, SinoMed, China National Knowledge Infrastructure (CNKI), China Science and Technology Periodical Database (VIP), and Wanfang Database. The objective was to identify randomized controlled trials (RCTs) evaluating HBOT’s effectiveness in alleviating sleep disorder symptoms in PD patients as an adjunct therapy. Literature screening and data extraction were independently executed by the authors. Meta-analyses were performed using Review Manager 5.3 software, and publication bias and sensitivity analyses were assessed using Stata 17.0 software.

**Results:**

Seven RCTs involving 461 participants were included. The findings revealed that the addition of HBOT significantly enhanced sleep efficiency (MD = 15.26, 95% CI [10.89, 19.63], *p* < 0.00001), increased time in bed (MD = 69.65, 95% CI [43.01, 96.30], *p* < 0.00001), total sleep time (MD = 75.87, 95% CI [25.42, 126.31], *p* = 0.003), slow-wave sleep (SWS) time (MD = 6.14, 95% CI [3.95, 8.34], *p* < 0.00001), and rapid eye movement sleep (REM) time (MD = 4.07, 95% CI [2.05, 6.08], *p* < 0.0001), and reduced awakening frequency (MD = −11.55, 95% CI [−15.42, −7.68], *p* < 0.00001) and sleep latency (MD = −6.60, 95% CI [−9.43, −3.89], *p* < 0.00001). Additionally, significant improvements were observed in the Pittsburgh Sleep Quality Index (PSQI) (MD = −2.52, 95% CI [−2.85, −2.18], *p* < 0.00001), Epworth Sleepiness Scale (ESS) (MD = −2.90, 95% CI [−3.34, −2.47], *p* < 0.00001), Unified Parkinson’s Disease Rating Scale Part III (UPDRS III) (MD = −1.32, 95% CI [−2.16, −0.47], *p* = 0.002), and Hoehn and Yahr grading (H-Y grading) (MD = −0.15, 95% CI [−0.28, −0.01], *p* = 0.03).

**Conclusion:**

The current meta-analysis supports the efficacy of HBOT as an adjunct therapy in managing sleep disorders in PD patients. It is recommended for PD patients experiencing sleep disturbances.

**Systematic review registration:**https://www.crd.york.ac.uk/, identifier: CRD42023462201.

## 1 Introduction

Sleep disorders are a common non-motor symptom in PD, affecting 60 to 90% of patients (1). Polysomnography (PSG) indicates that PD patients experience various types and degrees of sleep disorders, including insomnia, restless legs syndrome, rapid eye movement sleep behavior disorder, excessive daytime sleepiness, obstructive sleep apnea, and circadian rhythm disorders (2). These disorders can appear at any stage of PD, sometimes manifesting as the initial symptom. As the disease progresses, sleep disorders may interact with other motor and non-motor symptoms, leading to fatigue, anxiety, depression, and cognitive decline, significantly affecting patients’ overall well-being and productivity (3, 4).

The pathogenesis of PD-related sleep disorders is not fully understood, but it is believed to involve changes in neural anatomy, neuron loss, neurotransmitter alterations, genetics, environmental factors, and psychological influences (5). Treatment typically involves adjusting antiparkinsonian medications and incorporating sedative-hypnotics, which show some efficacy. However, optimizing the balance between controlling motor symptoms and sleep disturbances using antiparkinsonian drugs poses challenges. Moreover, the use of sedative-hypnotics is associated with potential adverse reactions and variable effectiveness that require further investigation (6).

HBOT, involving the therapeutic administration of 100 percent oxygen at pressures above 1.4 atmospheres, has shown promise in improving neurological conditions and neurodevelopmental disorders in both animal models and human studies (7, 8). Clinical reports indicate that HBOT may effectively treat sleep disorders in PD patients, supporting its potential as an adjunct treatment. Given the scarcity of large-scale, multi-center, randomized clinical trials, this meta-analysis seeks to review existing RCTs on HBOT for sleep disorders in PD to enhance the reliability of evidence-based clinical practices.

## 2 Materials and methods

### 2.1 Literature search

The databases searched by two authors included PubMed, Embase, Web of Science, Cochrane Library, China National Knowledge Infrastructure (CNKI), Wanfang Database, Chinese Science and Technology Periodical Database (VIP), and SinoMed. Keywords employed were “hyperbaric oxygenation,” “hyperbaric oxygen therapy,” “Parkinson’s disease,” “Idiopathic Parkinson’s disease,” “dyssomnias,” and “sleep disorders” (Supplementary Table S1). The search yielded all RCTs published up to September 2023.

### 2.2 Study selection and data extraction

Inclusion criteria were as follows: (1) RCTs reported in English or Chinese; (2) study populations clinically diagnosed with PD according to the “Clinical Diagnostic Criteria for Parkinson’s Disease” by the International Parkinson and Movement Disorder Society (MDS) (9) or the “Diagnostic Criteria for Parkinson’s Disease in China” by the Neurology Branch of the Chinese Medical Association (10) or other recognized diagnostic standards for PD. Additionally, diagnoses of sleep disorders were based on the “International Classification of Sleep Disorders, Third Edition” (ICSD-3) by the American Academy of Sleep Medicine (AASM) (11); (3) comparisons of HBOT plus standard treatment as recommended by guidelines versus standard treatment alone, or HBOT plus standard treatment versus placebo HBOT plus standard treatment; (4) studies reporting outcome measures relevant to symptoms of sleep disorders.

Exclusion criteria included: (1) dissertations; (2) duplicate publications; (3) animal experiments; (4) review articles; (5) interventions not pertinent to the study focus; (6) meta-analyses. Literature screening was independently conducted by two researchers using predefined criteria. Customized data extraction tables facilitated the collection of data, including general trial characteristics (first author’s name, publication date, and study period); baseline patient and disease information (sample size, age, and disease duration); interventions (treatment name, treatment dose, and duration); and definitions of outcomes. Discrepancies were resolved by consensus or involving a third researcher.

### 2.3 Quality assessment

The Cochrane risk of bias assessment tool was employed to evaluate the quality of RCTs, focusing on the following criteria: random sequence generation, concealment of the allocation sequence, blinding of participants and personnel, blinding of outcome assessment, completeness of outcome data, selective reporting, and other potential sources of bias (12). Risks within each category of bias were classified as low, high, or unclear. Discrepancies were resolved through discussion.

### 2.4 Statistical analysis

Data extraction was processed and analyzed using Review Manager software (V.5.3). Continuous variables were presented as mean differences (MDs) with their corresponding 95% confidence intervals (CIs). A *p*-value of less than 0.05 was considered statistically significant. The I^2^ statistic was employed to evaluate the extent of heterogeneity among the studies. An I^2^ value of 50% or less indicated acceptable heterogeneity, prompting the use of a fixed-effects model for analysis (13, 14). Conversely, significant heterogeneity, defined as I^2^ exceeding 50%, necessitated the application of a random-effects model (13, 14). In cases of significant heterogeneity, subgroup or sensitivity analyses were conducted to investigate potential sources. Egger’s test was applied to assess publication bias, with a significance level set at *p* < 0.05.

## 3 Results

### 3.1 Study inclusion and characteristics

**Figure 1:**
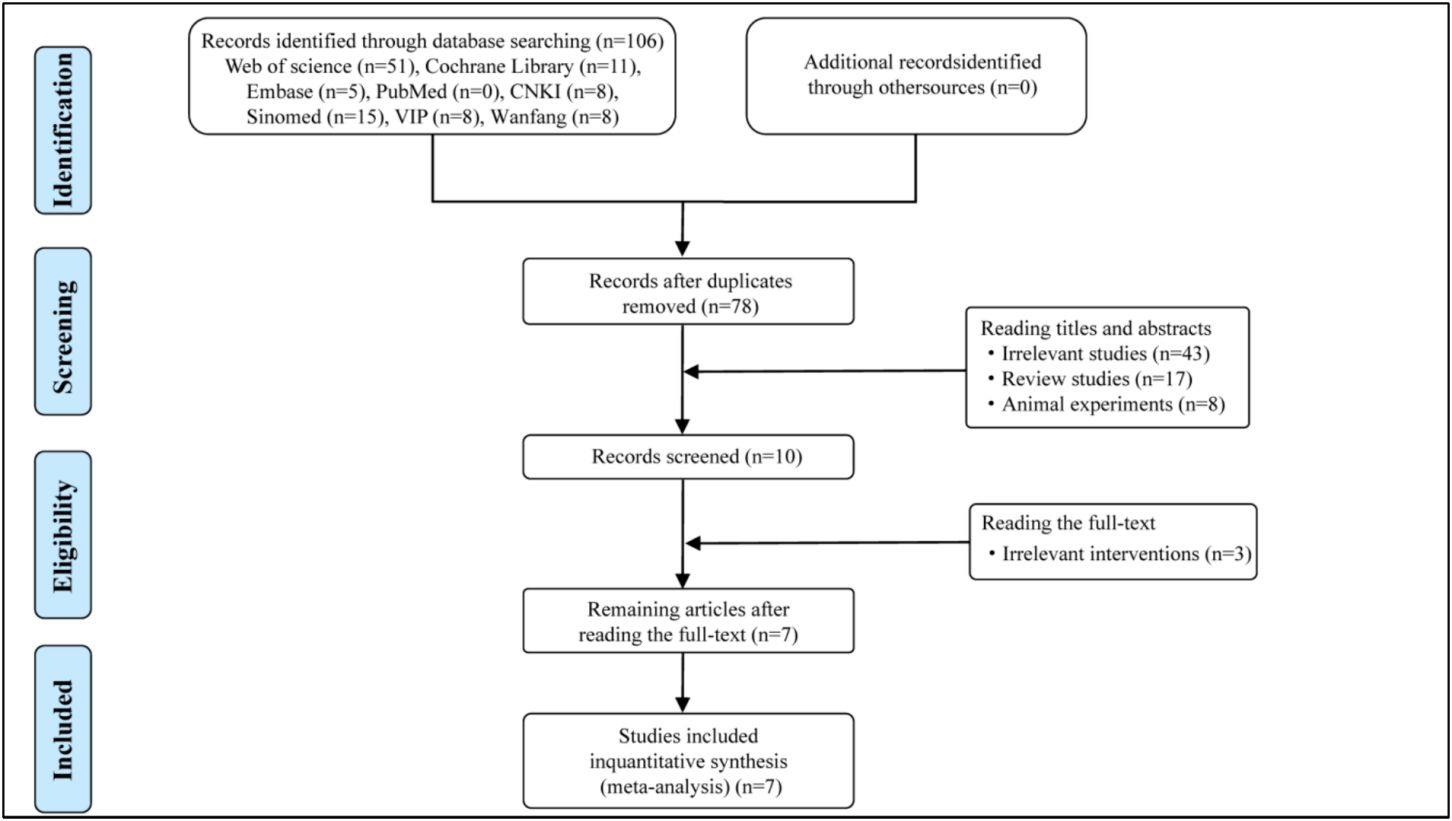
The flowchart of study selection.

A total of 106 potentially eligible articles were identified through database searches, from which 28 duplicate articles were removed. After screening the titles and abstracts, 10 articles were selected for full-text dual review, following the exclusion of 68 articles based on inclusion criteria. Of these, 3 articles were excluded, with detailed reasons provided in the flow diagram (Figure 1). Ultimately, 7 articles were included in this meta-analysis. The specific screening procedure is summarized in Figure 1. The included studies involved 461 participants comprising 233 participants in the experimental group and 228 participants in the control group. All comparisons involved HBOT plus Pramipexole versus Pramipexole alone. The treatment duration for all studies lasted 2 months with sample sizes varying from 40 to 128. Details regarding the study characteristics are presented in Table 1.

**Table 1:** Basic characteristics of the included studies.

First author, year	Reference	Study period	Sample size (E/C)	Gender (male/female)/n		Age (y)	Disease duration (y)	Hoehn-Yahr	Intervention measure		Treatment duration (m)	Evaluating indicator
				E	C				E	C		
Zheng HH, 2022	Zheng et al. (15)	2015–2016	31/31	17/14	16/15	E: 58.90 ± 5.70\C: 58.70 ± 5.30	T: 3.70 ± 1.00\C: 3.40 ± 1.10	≤ Level 3	HBO (60 min, bid, 0.2 MPa) + Pramipexole (0.5 mg tid PO)	Pramipexole (0.5 mg tid PO)	2	①②③④⑤⑥⑦⑧⑨⑩⑪⑫⑬
Luan H, 2021	Luan and Fu (16)	2017–2019	49/49	24/26	27/22	E: 60.82 ± 3.24\C: 60.31 ± 3.13	NR	≤ Level 3	HBO (60 min, bid, 0.2 MPa) + Pramipexole (0.5 mg tid PO)	Pramipexole (0.5 mg tid PO)	2	①②③④
Li HL, 2018	Li (17)	2014–2018	28/28	17/11	16/12	E: 65.43 ± 3.67\C: 65.47 ± 3.71	NR	NR	HBO (60 min, bid, 0.2 MPa) + Pramipexole (0.5 mg tid PO)	Pramipexole (0.5 mg tid PO)	2	②③④
Liu YY, 2018	Liu et al. (18)	2014–2016	45/45	23/22	26/19	E: 66.97 ± 12.53\C: 62.78 ± 11.79	T: 3.78 ± 1.74\C: 3.45 ± 1.31	≤ Level 3	HBO (60 min, bid, 0.2 MPa) + Pramipexole (0.5 mg tid PO)	Pramipexole (0.5 mg tid PO)	2	①②③④⑤⑥⑦⑧⑨⑩⑪⑫⑬
Wang GJ, 2017	Wang (19)	2015–2016	30/25	18/12	14/11	E:63.50 ± 6.70\C: 62.50 ± 6.60	T: 3.50 ± 1.70\C: 3.20 ± 1.50	≤ Level 3	HBO (60 min, bid, 0.2 MPa) + Pramipexole (0.5 mg tid PO)	Pramipexole (0.5 mg tid PO)	2	①②③④⑥⑦⑨⑩⑪⑫⑬
Pan Y, 2016	Pan et al. (20)	2012–2014	20/20	9/11	12/8	E: 61.35 ± 11.60\C: 60.10 ± 10.23	T: 3.48 ± 2.62\C: 3.05 ± 2.12	≤ Level 3	HBO (60 min, bid, 0.2 MPa) + Pramipexole (0.5 mg tid PO)	Pramipexole (0.5 mg tid PO)	2	①②③④⑤⑥⑦⑧⑨⑩⑪⑫⑬
Liu YX, 2016	Liu (21)	2013–2015	30/30	15/15	17/13	E: 61.75 ± 11.18\C: 61.18 ± 10.54	T: 3.69 ± 1.93\C: 3.26 ± 2.13	≥ Level 3	HBO (60 min, bid, 0.2 MPa) + Pramipexole(0.5 mg tid PO)	Pramipexole (0.5 mg tid PO)	2	①②③④⑤⑥⑦⑧⑨⑩⑪⑫⑬

E, Experimental group; C, Control group; HBO, Hyperbaric oxygen; NR, No record; PO, per os; ①, Sleep efficiency; ②, Bed time; ③, Total sleep time; ④, Awakening frequency; ⑤, Sleep latency; ⑥, Sleep time at phase I; ⑦, Sleep time at phase II; ⑧, Slow-wave sleep (SWS) time; ⑨, Rapid eye movement sleep (REM) time; ⑩, Pittsburgh Sleep Quality Index (PSQI); ⑪, Epworth sleepiness scale (ESS); ⑫, Unified Parkinson Disease Rating Scale III (UPDRS-III); ⑬, Hoehn-Yahr grading (H-Y grading).

### 3.2 Methodological quality assessment

**Figure 2:**
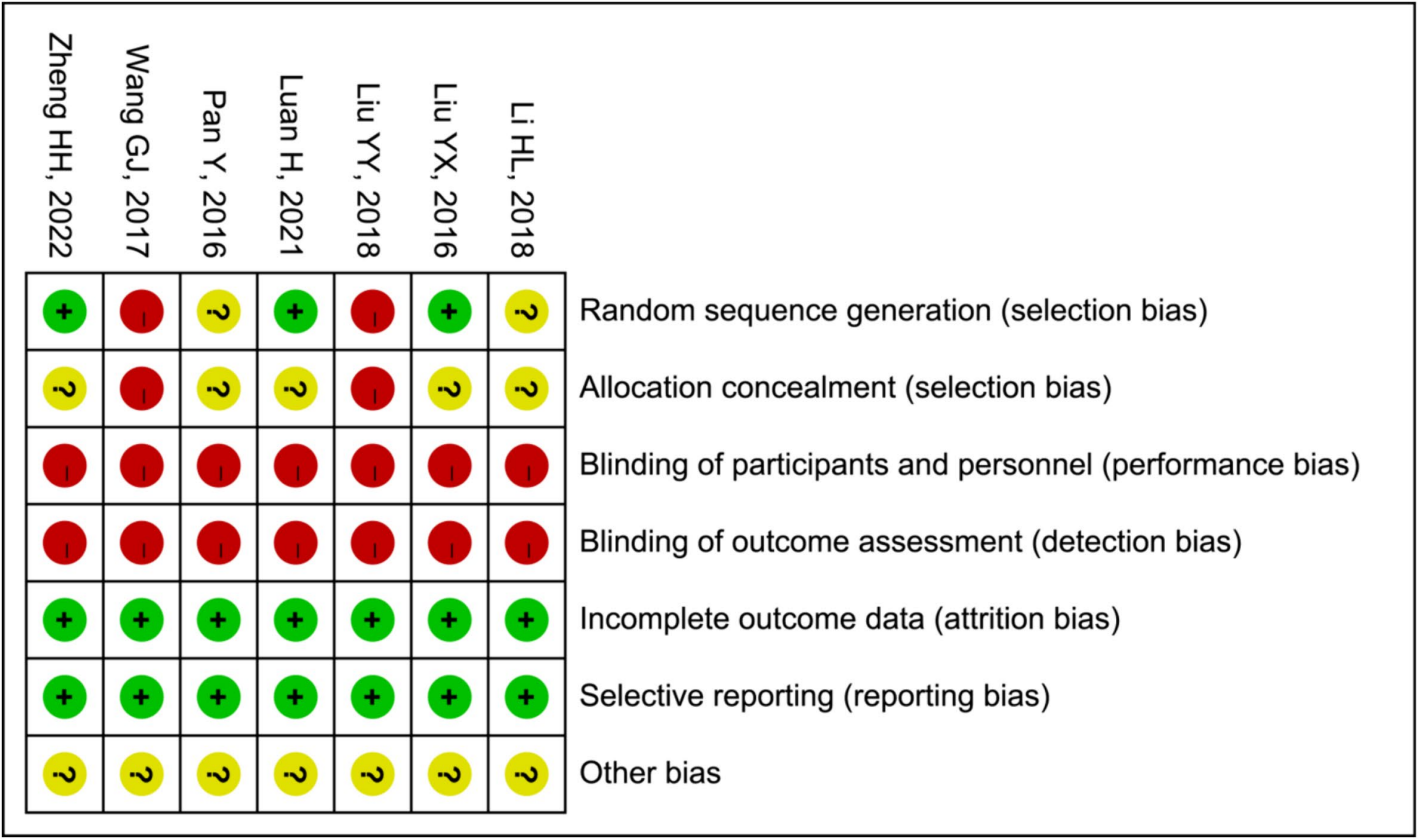
Risk of bias assessment of the 7 trials.

In two (15, 21) of the seven trials that were considered, random number tables were used to generate the sequences, while one study (16) employed a blind sampling method for grouping. Therefore, we deemed them to possess a low risk of bias. Four other studies (17, 19, 20, 22) did not disclose any information about the methodology employed for generating random sequences. Although all of the studies provided comprehensive data and various outcome indicators, they failed to specify allocation concealment, performance bias or detection bias. In summary, the quality of the literature is comparatively low or equivocal due to a significant portion of their research being devoted to biases. The specific outcomes derived from the bias assessment are summarized in Figure 2.

### 3.3 Effects of interventions

#### 3.3.1 Sleep efficiency

**Figure 3:**
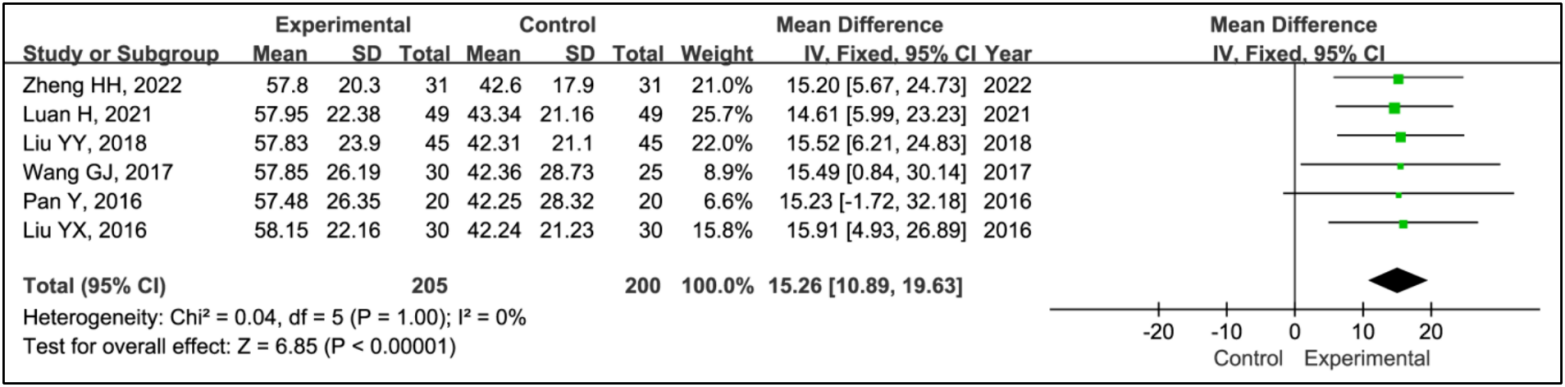
Forest plot of mean difference (MD) in sleep efficiency.

All included articles, except for one study (17), used sleep efficiency to evaluate treatment effects. This analysis incorporated data from 205 patients in experimental groups and 200 in control groups. Results indicated a significant improvement in sleep efficiency in the experimental group compared to the control group (MD = 15.26, 95% CI [10.89, 19.63], *p* < 0.00001), with no evidence of heterogeneity (*I^2^* = 0%, *p* = 1.00) (Figure 3).

#### 3.3.2 Time in bed

**Figure 4:**
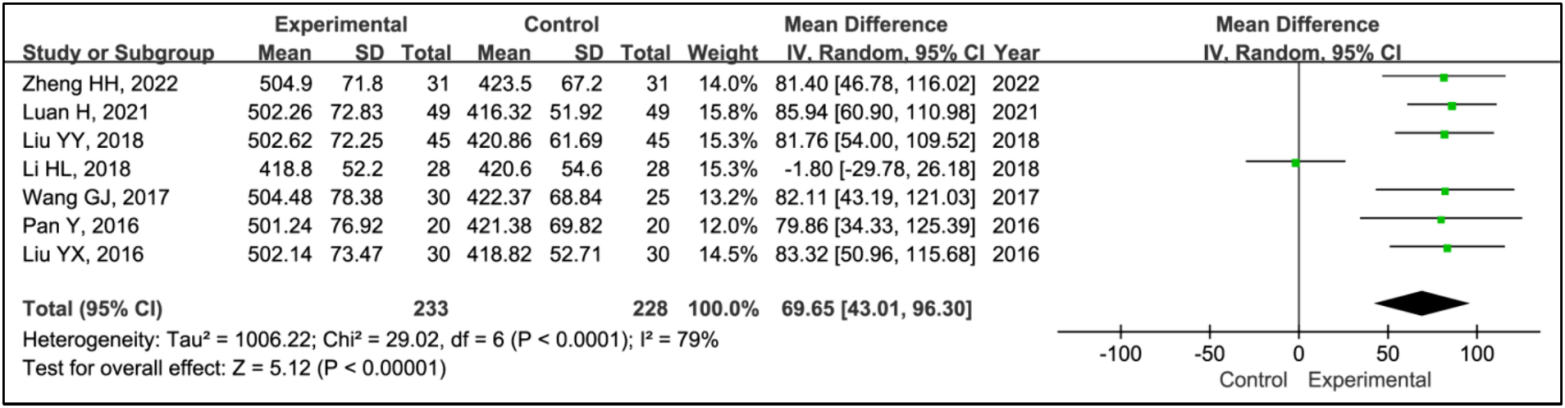
Forest plot of mean difference (MD) in time in bed.

All included studies used time in bed as a measure to evaluate treatment effects. This analysis encompassed data from 233 patients in experimental groups and 228 in control groups. The results showed a reduction in time in bed for the experimental group compared to the control group (MD = 69.65, 95% CI [43.01, 96.30], *p* < 0.00001), with significant heterogeneity observed (*I^2^* = 79%, *p* < 0.0001) (Figure 4). Due to this heterogeneity, a sensitivity analysis was conducted (Supplementary Figure S1). Following the exclusion of the article (17), heterogeneity among the studies was eliminated (*I^2^* = 0%, *p* = 1.00), and the outcomes remained consistent, indicating a stable effect size (MD = 82.99, 95% CI [69.89, 96.09], *p* < 0.00001) (Figure 4).

#### 3.3.3 Total sleep time

**Figure 5:**
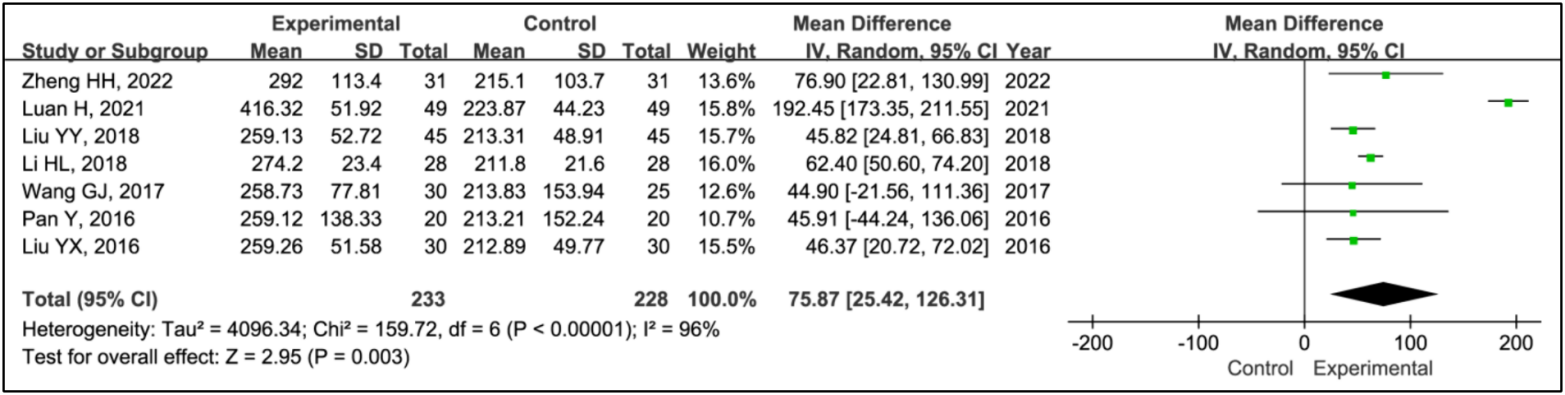
Forest plot of mean difference (MD) in total sleep time.

All studies assessed treatment effects using total sleep time. Data from 233 patients in experimental groups and 228 in control groups were combined for this analysis. Results indicated a reduction in total sleep time for the experimental group compared to the control group (MD = 75.87, 95% CI [25.42, 126.31], *p* = 0.003), with significant heterogeneity observed (*I^2^* = 96%, *p* < 0.00001) (Figure 5). Due to this heterogeneity, a sensitivity analysis was performed (Supplementary Figure S1). After the exclusion of the article (16), heterogeneity among the studies was eliminated (*I^2^* = 0%, *p* = 0.66), and the outcomes remained consistent, showing a stable effect size (MD = 57.00, 95% CI [47.74, 66.26], *p* < 0.00001) (Figure 5).

#### 3.3.4 Awakening frequency

**Figure 6:**
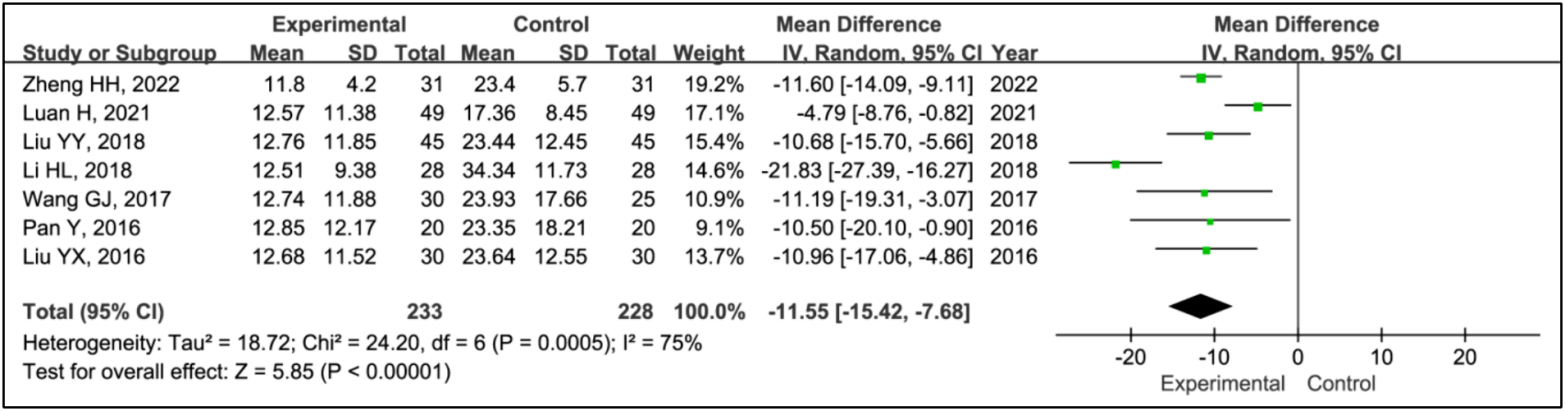
Forest plot of mean difference (MD) in awakening frequency.

All studies assessed treatment effects using awakening frequency. The analysis included data from 233 patients in experimental groups and 228 in control groups. It revealed a significant reduction in awakening frequency within the experimental group compared to the control group (MD = −11.55, 95% CI [−15.42, −7.68], *p* < 0.00001), with notable heterogeneity (*I^2^* = 75%, *p* = 0.0005) (Figure 6). Due to this heterogeneity, a sensitivity analysis was conducted (Supplementary Figure S1). After the exclusion of the article (17), heterogeneity was reduced to an acceptable level (*I^2^* = 41%, *p* = 0.13), and the outcomes remained consistent, indicating a stable effect size (MD = −10.01, 95% CI [−11.79, −8.23], *p* < 0.00001) (Figure 6).

#### 3.3.5 Sleep latency

**Figure 7:**
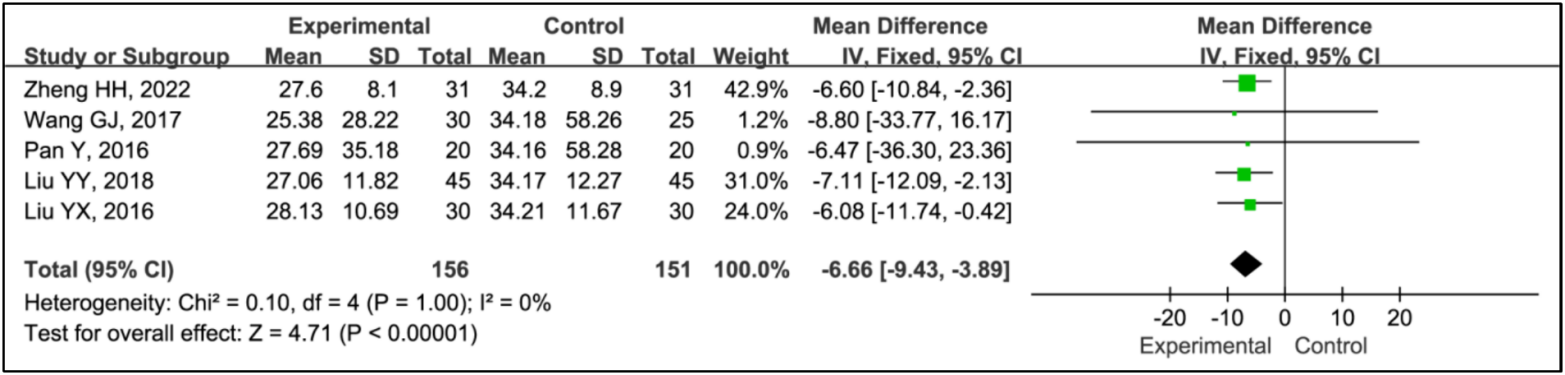
Forest plot of mean difference (MD) in sleep latency.

Five studies assessed treatment effects using sleep latency (15, 19–22). Data from 156 patients in experimental groups and 151 in control groups were analyzed. The results indicated a significant reduction in sleep latency between the experimental and control groups (MD = −6.60, 95% CI [−9.43, −3.89], *p* < 0.00001), with no evidence of heterogeneity (*I^2^* = 0%, *p* = 0.98) (Figure 7).

#### 3.3.6 Sleep time during phase I

**Figure 8:**
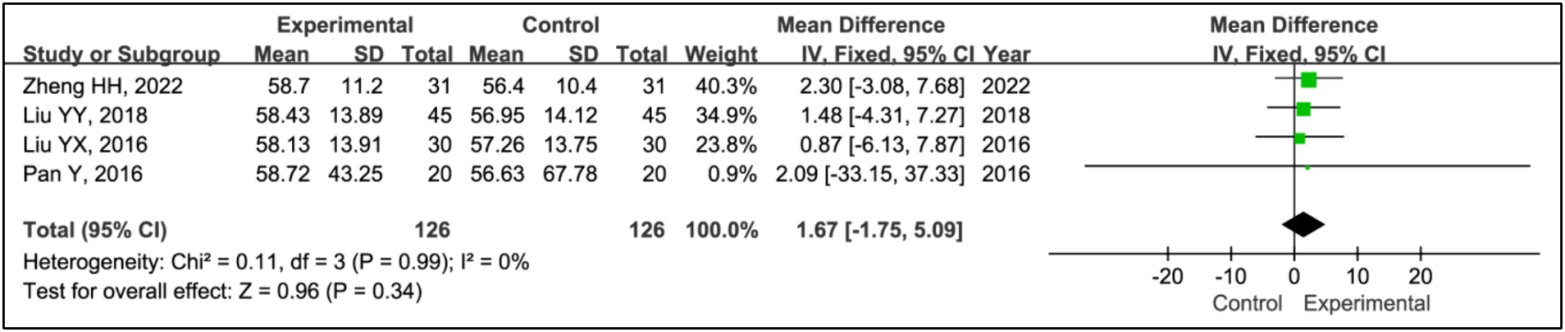
Forest plot of mean difference (MD) in sleep time during phase I.

Four studies employed sleep time during phase I to evaluate treatment effects (15, 20–22). This research included data from 126 patients in experimental groups and 126 in control groups. The analysis indicated no significant differences in phase I sleep time between the experimental and control groups (MD = 1.67, 95% CI [−1.75, 5.09], *p* = 0.34), with acceptable heterogeneity (*I^2^* = 0%, *p* = 0.99) (Figure 8).

#### 3.3.7 Sleep time during phase II

**Figure 9:**
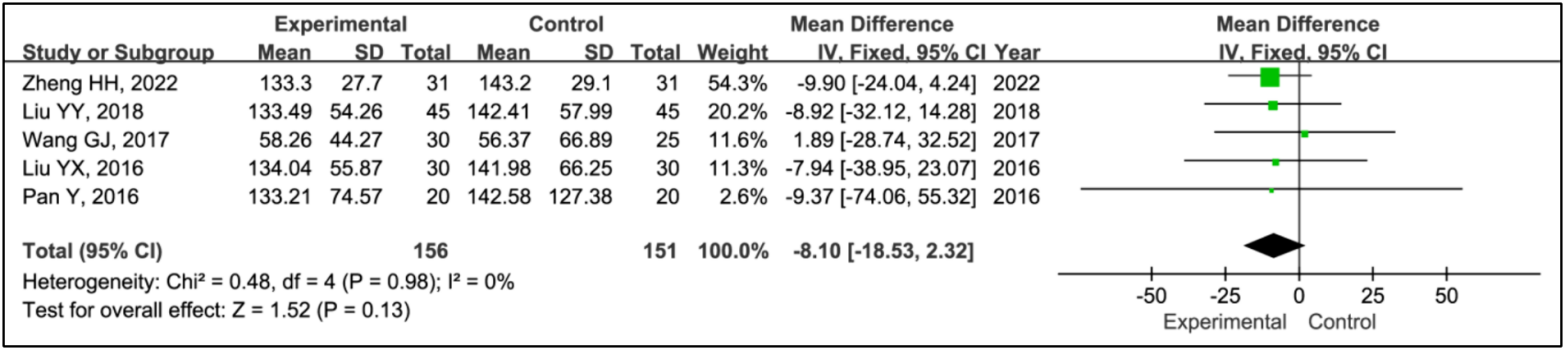
Forest plot of mean difference (MD) in sleep time during phase II.

Five studies assessed treatment effects using sleep time during phase II (15, 19–22). Data from 156 patients in experimental groups and 151 in control groups were analyzed. The results indicated no significant differences in phase II sleep time between the experimental and control groups (MD = −8.10, 95% CI [−18.53, 2.32], *p* = 0.13), with no evidence of heterogeneity (*I^2^* = 0%, *p* = 0.98) (Figure 9).

#### 3.3.8 SWS time

**Figure 10:**
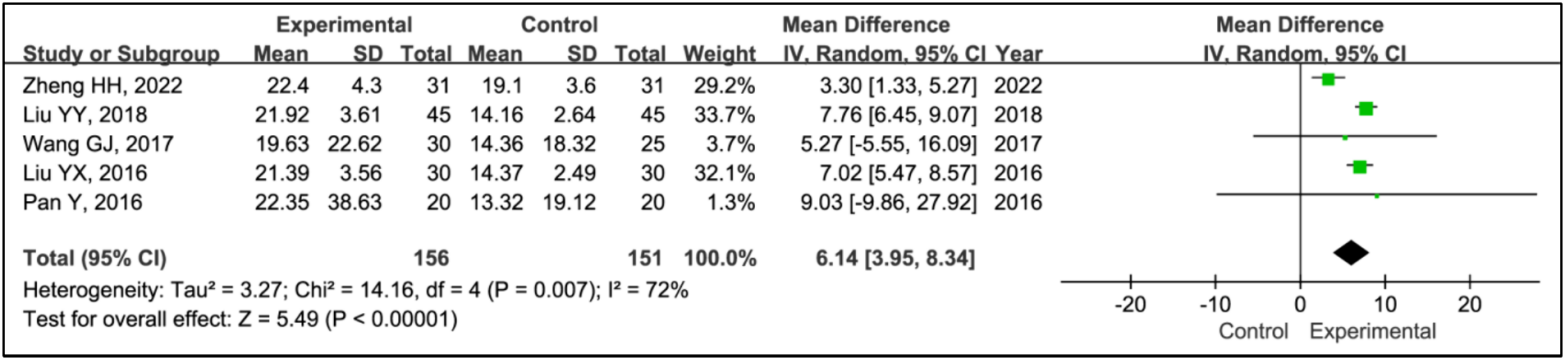
Forest plot of mean difference (MD) in SWS time.

Five studies assessed the effects of treatments on slow wave sleep (SWS) time (15, 19–22). The analysis included data from 156 patients in experimental groups and 151 in control groups. It showed a significant increase in SWS time in the experimental group compared to the control group (MD = 6.14, 95% CI [3.95, 8.34], *p* < 0.00001), with substantial heterogeneity (*I^2^* = 0%, *p* = 0.87) (Figure 9). Due to this heterogeneity, a sensitivity analysis was conducted (Supplementary Figure S1). Removing the study (15) eliminated heterogeneity (*I^2^* = 0%, *p* = 0.87), and the outcomes remained consistent, indicating a stable effect size (MD = 7.44, 95% CI [6.44, 8.43], *p* < 0.00001) (Figure 10).

#### 3.3.9 REM time

**Figure 11:**
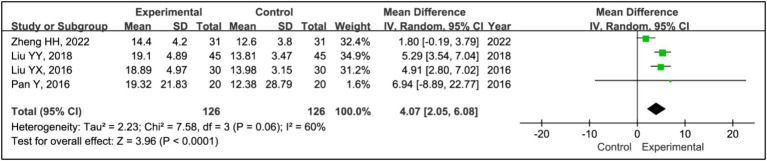
Forest plot of mean difference (MD) in REM time.

Four studies utilized rapid eye movement sleep (REM) time to evaluate treatment effects (15, 20–22). This research aggregated data from 126 patients in the experimental groups and 126 patients in the control groups. The analysis showed a significant increase in REM time in the experimental group compared to the control group (MD = 4.07, 95% CI [2.05, 6.08], *p* < 0.0001), with notable heterogeneity (*I^2^* = 60%, *p* = 0.06) (Figure 10). Due to this heterogeneity, a sensitivity analysis was conducted (Supplementary Figure S1). With the removal of the study (15), heterogeneity was reduced to acceptable levels (*I^2^* = 0%, *p* = 0.94), and the outcomes were consistent, indicating a stable effect size (MD = 5.15, 95% CI [3.81, 6.49], *p* < 0.00001) (Figure 11).

#### 3.3.10 PSQI

**Figure 12:**
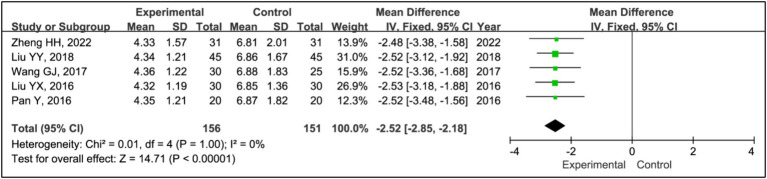
Forest plot of mean difference (MD) in PSQI score.

Five studies utilized the Pittsburgh Sleep Quality Index (PSQI) to assess treatment effects (15, 19–22). This research included data from 156 patients in experimental groups and 151 in control groups. The analysis demonstrated a significant improvement in PSQI scores within the experimental group compared to the control group (MD = −2.52, 95% CI [−2.85, −2.18], *p* < 0.00001), with no observed heterogeneity (*I^2^* = 0%, *p* = 1.00) (Figure 12).

#### 3.3.11 ESS

**Figure 13:**
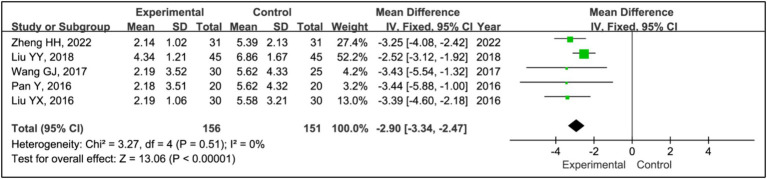
Forest plot of mean difference (MD) in ESS score.

Five studies employed the Epworth Sleepiness Scale (ESS) to assess treatment effects (15, 19–22). This research integrated data from 156 patients in experimental groups and 151 in control groups. The analysis indicated a significant decrease in ESS scores within the experimental group compared to the control group (MD = −2.90, 95% CI [−3.34, −2.47], *p* < 0.00001), with no observed heterogeneity (*I^2^* = 0%, *p* = 0.51) (Figure 13).

#### 3.3.12 UPDRS III

**Figure 14:**
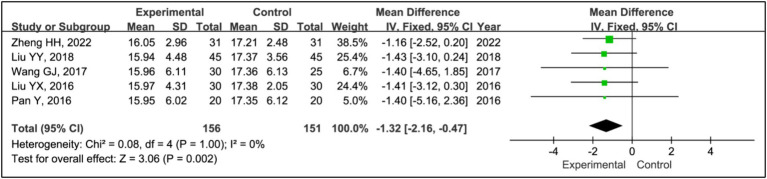
Forest plot of mean difference (MD) in UPDRS III score.

Five studies utilized the Unified Parkinson’s Disease Rating Scale Part III (UPDRS III) to assess treatment effects (15, 19–22). This analysis included data from 156 patients in experimental groups and 151 in control groups. It showed a significant reduction in UPDRS III scores within the experimental group compared to the control group (MD = −1.32, 95% CI [−2.16, −0.47], *p* = 0.002), with no observed heterogeneity (*I^2^* = 0%, *p* = 1.00) (Figure 14).

#### 3.3.13 H-Y grading

**Figure 15:**
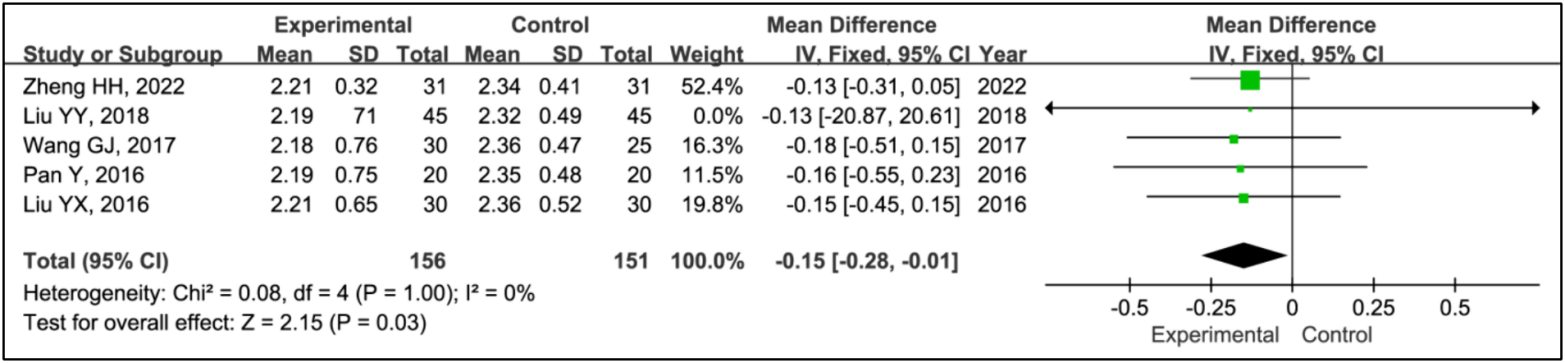
Forest plot of mean difference (MD) in H-Y grading score.

Five studies utilized the Hoehn and Yahr (H-Y) grading system to evaluate treatment effects (15, 19–22). This analysis incorporated data from 156 patients in experimental groups and 151 in control groups. The results indicated a significant improvement in H-Y grading within the experimental group compared to the control group (MD = −0.15, 95% CI [−0.28, −0.01], *p* = 0.03), with no observed heterogeneity (*I^2^* = 0%, *p* = 1.00) (Figure 15).

#### 3.3.14 Sensitivity analysis

Stata 17.0 software was employed to perform a comprehensive sensitivity analysis on the primary outcomes of our study, which included time in bed, total sleep time, awakening frequency, SWS time and REM time. The analysis showed that the exclusion of any single study from each outcome except SWS time did not significantly alter the overall results, thereby confirming the robustness and reliability of this meta-analysis (Supplementary Figures S2–S6).

#### 3.3.15 Publication bias

We assessed potential publication bias in primary outcomes using Stata 17.0 and Egger’s test. The results indicated no significant publication bias across various parameters: sleep efficiency (*p* = 0.486), time in bed (*p* = 0.679), total sleep time (*p* = 0.953), awakening frequency (*p* = 0.769), sleep latency (*p* = 0.646), sleep time during phase I (*p* = 0.868), sleep time during phase II (*p* = 0.452), SWS time (*p* = 0.738), REM time (*p* = 0.933), PSQI (*p* = 0.357), ESS (*p* = 0.182), UPDRS III (*p* = 0.435), and H-Y grading (*p* = 0.095). These findings suggest a high level of integrity in the reported data.

## 4 Discussion

### 4.1 Summary of main findings

Sleep disorders are a fundamental aspect of PD, and managing them presents significant challenges. This study incorporated seven trials to assess whether HBOT serves as an effective adjunct therapy for PD-related sleep disorders.

PSG, a diagnostic technique with multiple leads and parameters, has been widely applied in sleep medicine since Jerome Holland refined its nomenclature in 1974. Owing to its comprehensive recording capabilities and exceptional reliability, PSG plays a crucial role in the foundational assessment of sleep disorders (22). Utilizing data from channels such as the electroencephalogram and electrooculogram, PSG can accurately compute sleep structural parameters, including sleep latency, total sleep time, and sleep efficiency. It not only objectively assesses sleep structure and related indicators but also reduces evaluation bias inherent in subjective assessments (22). Objective testing using PSG has shown that common alterations in the sleep architecture of individuals with PD include reductions in total sleep duration, frequent arousals, and overall fragmentation of sleep (23). This meta-analysis revealed that the experimental group significantly outperformed the control group in terms of sleep efficiency, time in bed, total sleep time, sleep latency, SWS time, and REM time. The results indicated that HBOT combined with pramipexole could improve PD patients’ sleep parameters. This suggests that HBOT combined with drug therapy can enhance the sleep architecture of PD patients with sleep disorders, thereby improving their sleep quality.

Subjective evaluation indicators in this study included the PSQI, ESS, UPDRS III, and H-Y grading. The PSQI is utilized to assess sleep quality (24), and the ESS measures daytime drowsiness (25). UPDRS III evaluates motor function (26), while H-Y grading assesses the progression of Parkinson’s disease (27). This meta-analysis demonstrated that patients in the experimental group experienced greater improvements in PSQI and ESS scores compared to those in the control group. Furthermore, compared to patients who only underwent therapy with pramipexole, those who received HBOT in combination also showed substantial improvements in UPDRS III scores and H-Y grading. The results suggested that integrating HBOT with drug therapy could reduce daytime drowsiness, enhance sleep stability, and potentially improve motor function, delay or even reverse the progression of the disease.

### 4.2 Mechanisms of HBOT

Several prior studies have demonstrated the efficacy of HBOT with or without drug in treating PD and associated sleep disturbances, exploring its underlying mechanisms. Research involving animals indicated that mild hyperbaric oxygen could prevent the death of dopaminergic neurons in the substantia nigra of mice with PD induced by 1-methyl-4-phenyl-1,2,3,6-tetrahydropyridine (MPTP) (28). Further, another study confirmed that HBOT could improve PD by stimulating mitochondrial biogenesis through the SIRT-1/PGC-1α pathway (29). Oxidative stress, pivotal in PD pathogenesis, involves damage to dopamine neurons. Pathologically, a marked reduction in antioxidant agents in the substantia nigra leads to an accumulation of free radicals, triggering oxidative stress. This stress is linked to the accumulation of cytotoxic compounds, enzyme failures, lipid peroxide formation, and ultimately, the death of substantia nigra cells, contributing to PD progression (30). A research reported that elevated serum levels of glutathione peroxidase (GSH-Px), malondialdehyde (MDA), and superoxide dismutase (SOD) in patients with insomnia compared to healthy individuals, suggesting a role for oxidative stress in the pathogenesis of insomnia (31). Consequently, oxidative stress may also contribute to PD associated with sleep disorders. Wang et al. (32) divided 80 PD patients into treatment groups and found that combining HBOT with standard dopamine therapy significantly improved Unified Parkinson’s Disease Rating Scale (UPDRS) and Parkinson’s Disease Sleep Scale (PDSS) scores, reduced serum MDA levels, and increased serum SOD and GSH-Px levels. Similarly, Chen et al. (33) demonstrated that HBOT combined with selegiline further reduced serum MDA levels and enhanced serum SOD levels, thereby improving motor functions and sleep quality in PD patients with sleep disorders. Additionally, Li et al. (34) noted that reduced brain-derived neurotrophic factor (BDNF) expression is linked to the deterioration of dopaminergic neurons in the substantia nigra and the progression of PD, with decreased BDNF secretion also contributing to sleep disorders. This decrease in BDNF is associated with a reduction in γ-aminobutyric acid (GABA), leading to heightened cerebral cortex excitability and subsequent sleep disturbances. This mechanism could explain the sleep disorders observed in PD patients (35). A research (36) found that HBOT combined with selegiline not only improved sleep architecture and quality in PD patients but also increased GABA levels and altered glutamate (Glu)/GABA ratios, highlighting the potential of HBOT combined with drug therapy to mitigate sleep-related symptoms in PD.

### 4.3 Study limitations

This study provides a valuable reference for clinical practice, yet it is essential to recognize its inherent limitations. Firstly, through rigorous literature screening, only seven articles were selected, all concerning the treatment of Chinese patients with PD. This narrow focus limits understanding of the efficacy of HBOT combined with drug therapy for sleep disorders in PD patients both domestically and internationally. Secondly, the included articles generally do not exhibit a high level of quality. Thirdly, it is crucial to acknowledge that the studies did not categorize the various types of sleep disorders present in individuals with PD, which restricts the ability to assess the effectiveness of HBOT combined with drug therapy across different sleep disorder categories. Finally, the safety assessment protocols in most included trials were found to be lacking.

## 5 Conclusion

This research supports the potential of HBOT combined with drug therapy to treat sleep disorders in PD patients by enhancing sleep quality, extending sleep duration, reducing daytime sleepiness, improving sleep structure, and preserving sleep stability. Additionally, they may improve motor function and possibly delay or even reverse disease progression in PD patients. Therefore, HBOT has the potential to be widely adopted as an adjunct therapy for PD associated with sleep disorders.

## Data availability statement

The original contributions presented in the study are included in the article/Supplementary material, further inquiries can be directed to the corresponding authors.

## Author contributions

W-qT: Formal analysis, Writing – original draft, Conceptualization, Data curation, Funding acquisition. QL: Data curation, Formal analysis, Investigation, Project administration, Supervision, Writing – review & editing. M-jC: Investigation, Software, Validation, Visualization, Writing – original draft. IL: Conceptualization, Investigation, Methodology, Writing – original draft. Z-qP: Writing – review & editing, Funding acquisition, Methodology, Project administration, Supervision. M-xL: Writing – review & editing, Data curation, Investigation. L-xZ: Formal analysis, Funding acquisition, Project administration, Writing – original draft.
