# Supplementary material for: Efficacy of hyperbaric oxygen therapy as an adjunct therapy in the treatment of sleep disorders among patients with Parkinson’s disease: a meta-analysis
Source: Front Neurol. 2024 Jul 31;15:1328911. doi: 10.3389/fneur.2024.1328911 (PMC11322060; doi:10.3389/fneur.2024.1328911)
Supplement: Supplementary file 1 [file Data_Sheet_1.docx]

Supplementary Material

Efficacy and safety of hyperbaric oxygen therapy in the treatment of sleep disorders of patients with Parkinson's disease: a meta-analysis

# 1 Supplementary **Table**

**Table 1.** The detailed search strategy.

**Search strategy for CNKI**

(TKA=‘高压氧’+‘高压氧疗法’) AND (TKA= ‘帕金森病’+‘帕金森’+‘震颤麻痹’) AND (‘睡眠’+‘睡眠障碍’+‘睡眠问题’+‘嗜睡’+‘不宁腿综合征’+‘阻塞性睡眠呼吸暂停’+‘昼夜节律’+‘失眠’+‘快速眼球运动睡眠期行为障碍’)

**Search strategy for Wanfang**

主题:(高压氧 OR 高压氧疗法) and 主题:(帕金森病 OR 帕金森 OR 震颤麻痹) and 主题:(睡眠 OR 睡眠障碍 OR 睡眠问题 OR 嗜睡 OR 不宁腿综合征 OR 阻塞性睡眠呼吸暂停 OR 昼夜节律 OR 失眠 OR 快速眼球运动睡眠期行为障碍)

**Search strategy for VIP**

题名或关键词=高压氧+高压氧疗法AND题名或关键词=帕金森+帕金森病+震颤麻痹AND题名或关键词=睡眠+睡眠障碍+睡眠问题+嗜睡+不宁腿综合征+阻塞性睡眠呼吸暂停+昼夜节律+失眠+快速眼球运动睡眠期行为障碍

**SinoMed**

(("帕金森"[常用字段:智能] OR "震颤麻痹"[常用字段:智能]) OR ("帕金森病"[不加权:扩展])) AND (("睡眠"[常用字段:智能] OR "睡眠问题"[常用字段:智能] OR "嗜睡"[常用字段:智能] OR "不宁腿综合征"[常用字段:智能] OR "阻塞性睡眠呼吸暂停"[常用字段:智能] OR "昼夜节律"[常用字段:智能] OR "失眠"[常用字段] OR "快速眼球运动睡眠期行为障碍"[常用字段:智能]) OR ("睡眠障碍"[不加权:扩展])) AND (("高压氧疗法"[常用字段:智能]) OR ("高压氧"[不加权:扩展]))

**Search strategy for PubMed**

| Search | Terms |
| --- | --- |
| #1 | "Hyperbaric Oxygenation"[MeSH Terms] |
| #2 | "hyperbaric oxygenations"[Title/Abstract] OR"hyperbaric oxygen therapy" OR"hyperbaric oxygen therapies"[Title/Abstract] OR "high pressure oxygen" [Title/Abstract] OR "oxygen therapy"[Title/Abstract] |
| #3 | "Parkinson Disease"[MeSH Terms] |
| #4 | "Parkinson's Disease"[Title/Abstract] OR "Idiopathic Parkinson's Disease"[Title/Abstract] OR "Idiopathic Parkinson Disease"[Title/Abstract] OR "Lewy Body Parkinson's Disease"[Title/Abstract] OR "Lewy Body Parkinson Disease"[Title/Abstract] OR "Primary Parkinsonism"[Title/Abstract] OR "Paralysis Agitans"[Title/Abstract] |
| #5 | "Dyssomnias"[MeSH Terms] |
| #6 | "dyssomnia"[Title/Abstract] OR "sleep disorders"[Title/Abstract] OR "sleep disturbances"[Title/Abstract] OR "sleep dysfunction"[Title/Abstract] OR "sleep" [Title/Abstract] OR "sleepiness"[Title/Abstract] OR "sleep problem" [Title/Abstract] OR "insomnia"[Title/Abstract] OR "restless legs syndrome" [Title/Abstract] OR "rapid eye movement sleep behavior disorder"[Title/Abstract] OR "excessive daytime sleepiness"[Title/Abstract] OR "obstructive sleep apnea" [Title/Abstract] OR "circadian rhythm"[Title/Abstract] |
| #7 | #1 OR #2 |
| #8 | #3 OR #4 |
| #9 | #5 OR #6 |
| #10 | #7 AND #8 AND #9 |

**Search strategy for Web of Science**

| Search | Terms |
| --- | --- |
| #1 | hyperbaric oxygenation (Topic) OR hyperbaric oxygen therapy (Topic) OR hyperbaric oxygenations (Topic) OR hyperbaric oxygen therapies (Topic) OR High pressure oxygen (Topic) OR Oxygen Therapy (Topic) |
| #2 | Parkinson Disease (Topic) OR Parkinson's Disease (Topic) OR Idiopathic Parkinson's Disease (Topic) OR Idiopathic Parkinson Disease (Topic) OR Lewy Body Parkinson's Disease (Topic) OR Lewy Body Parkinson Disease (Topic) OR Primary Parkinsonism (Topic) OR Paralysis Agitans (Topic) |
| #3 | dyssomnias (Topic) dyssomnia (Topic) OR sleep disorders (Topic) OR sleep disturbances (Topic) OR sleep dysfunction (Topic) OR sleep (Topic) OR sleepiness (Topic) OR sleep problem (Topic) OR insomnia (Topic) OR restless legs syndrome (Topic) OR rapid eye movement sleep behavior disorder (Topic) OR excessive daytime sleepiness (Topic) OR obstructive sleep apnea (Topic) OR circadian rhythm (Topic) |
| #4 | #1 AND #2 AND #3 |

**Search strategy for Embase**

| Search | Terms |
| --- | --- |
| #1 | 'hyperbaric oxygenation'/exp |
| #2 | 'hyperbaric oxygenations':ab,ti OR 'hyperbaric oxygen therapies':ab,ti OR 'hyperbaric oxygen therapy':ab,ti OR 'high pressure oxygen':ab,ti OR 'oxygen therapy':ab,ti |
| #3 | #1 OR #2 |
| #4 | 'Parkinson Disease'/exp |
| #5 | 'Parkinson's Disease':ab,ti OR 'Idiopathic Parkinson's Disease':ab,ti OR 'Idiopathic Parkinson Disease':ab,ti OR 'Lewy Body Parkinson's Disease':ab,ti OR 'Lewy Body Parkinson Disease':ab,ti OR 'Primary Parkinsonism':ab,ti OR 'Paralysis Agitans':ab,ti |
| #6 | #4 OR #5 |
| #7 | 'dyssomnias'/exp |
| #8 | 'dyssomnia':ab,ti OR 'sleep disorders':ab,ti OR 'sleep disturbances':ab,ti OR 'sleep dysfunction':ab,ti OR 'sleep':ab,ti OR 'sleepiness':ab,ti OR 'sleep problem':ab,ti OR 'insomnia':ab,ti OR 'restless legs syndrome':ab,ti OR 'rapid eye movement sleep behavior disorder':ab,ti OR 'excessive daytime sleepiness':ab,ti OR 'obstructive sleep apnea':ab,ti OR 'circadian rhythm':ab,ti |
| #9 | #7 OR #8 |
| #10 | #3 AND #6 AND #9 |

**Search strategy for Cochrane Library**

| Search | Terms |
| --- | --- |
| #1 | MeSH descriptor: [Hyperbaric Oxygenation] explode all trees |
| #2 | (hyperbaric oxygenations):ti,ab,kw OR (hyperbaric oxygen therapies):ti,ab,kw OR (hyperbaric oxygen therapy):ti,ab,kw OR (high pressure oxygen):ti,ab,kw OR (oxygen therapy):ti,ab,kw |
| #3 | #1 OR #2 |
| #4 | MeSH descriptor: [Parkinson Disease] explode all trees |
| #5 | (Parkinson's Disease):ti,ab,kw OR (Idiopathic Parkinson's Disease):ti,ab,kw OR (Idiopathic Parkinson Disease):ti,ab,kw OR (Lewy Body Parkinson's Disease):ti,ab,kw OR (Lewy Body Parkinson Disease):ti,ab,kw OR (Primary Parkinsonism):ti,ab,kw OR (Paralysis Agitans) |
| #6 | #4 OR #5 |
| #7 | MeSH descriptor: [Dyssomnias] explode all trees |
| #8 | (dyssomnia):ti,ab,kw OR (sleep disorders):ti,ab,kw OR (sleep disturbance) :ti,ab,kw OR (sleep dysfunction):ti,ab,kw OR (sleep):ti,ab,kw OR (sleepiness):ti,ab,kw OR (sleep problem):ti,ab,kw OR (insomnia):ti,ab,kw OR (restless legs syndrome):ti,ab,kw OR (rapid eye movement sleep behavior disorder):ti,ab,kw OR (excessive daytime sleepiness):ti,ab,kw OR (obstructive sleep apnea):ti,ab,kw OR (circadian rhythm):ti,ab,kw |
| #9 | #7 OR #8 |
| #10 | #3 AND #6 AND #9 |

# 2 Supplementary Figure 1


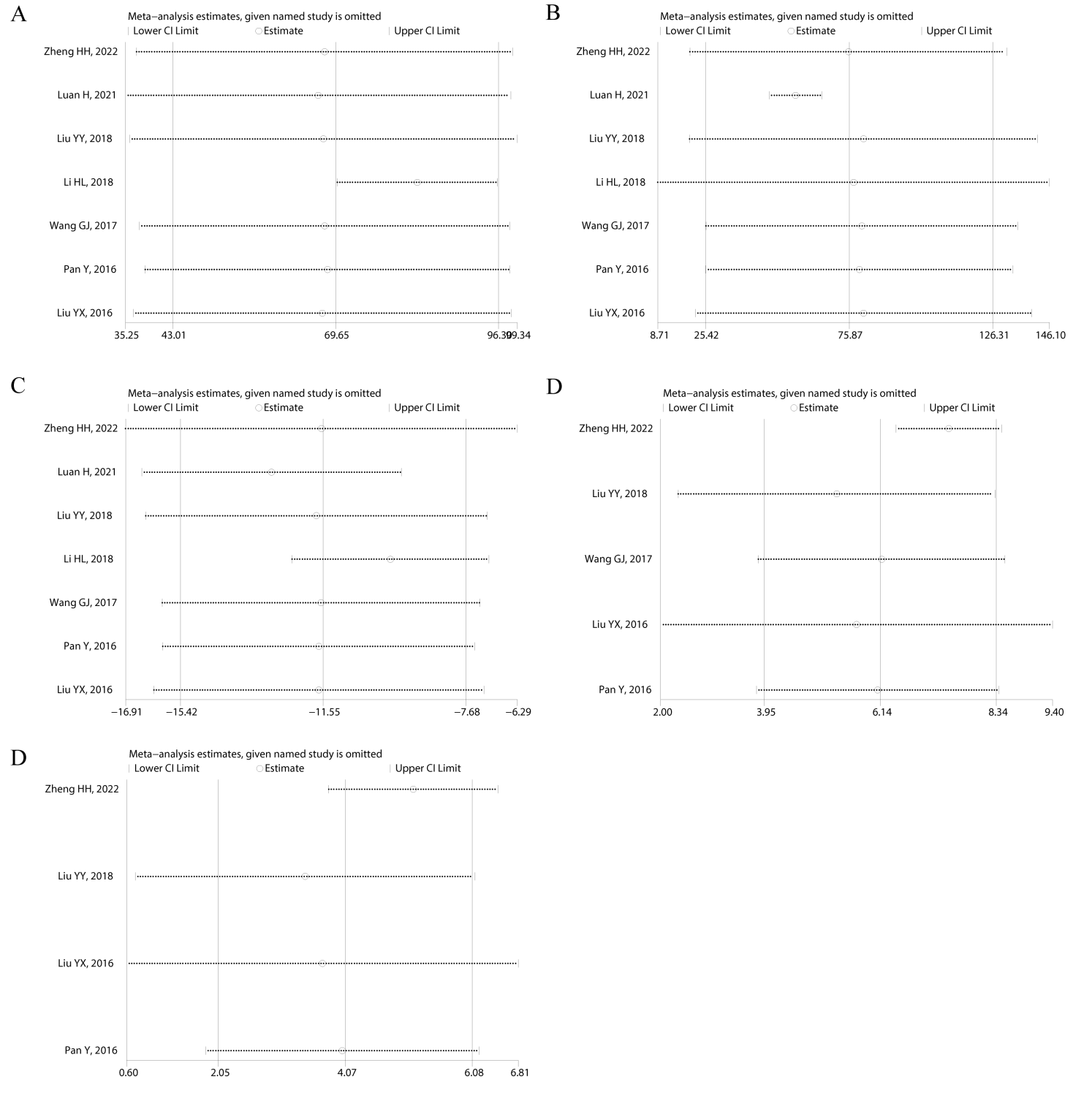


**Figure 1 Sensitivity analysis plot of (A) time in bed, (B) total sleep time, (C) awakening frequency, (D) SWS time and (E) REM time.**

# The supplementary figures 2-6 are all leave-one-out meta-analysis plot which were analysied by Stata 17.0. In meta-analysis, a leave-one-out plot is often used to assess the impact of individual studies on the overall meta-analysis results. The plot typically displays the results of a meta-analysis with all studies included. Each point on the plot represents the effect size (e.g., mean difference) of the meta-analysis when a particular study is left out. Points that cause substantial changes in the meta-analysis results when omitted may be considered influential or outliers. If removing a particular study consistently alters the results, it indicates that the study has a strong influence on the overall conclusion. Otherwise, the results of the meta-analysis are robust.


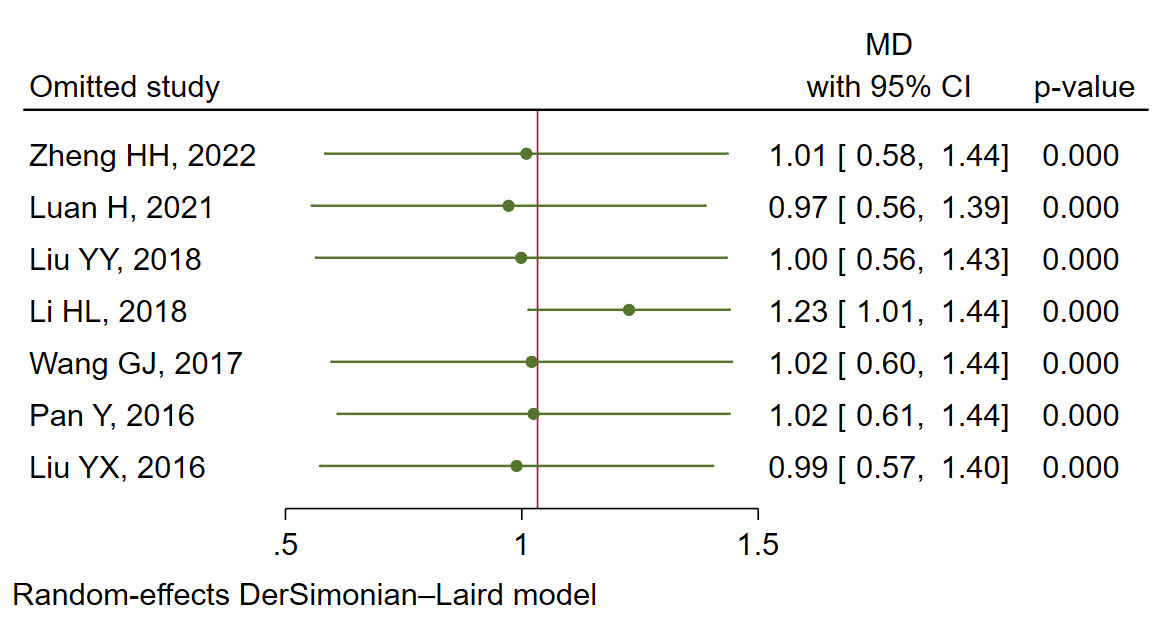


**Figure 2 Leave-one-out meta-analysis plot of time in bed**


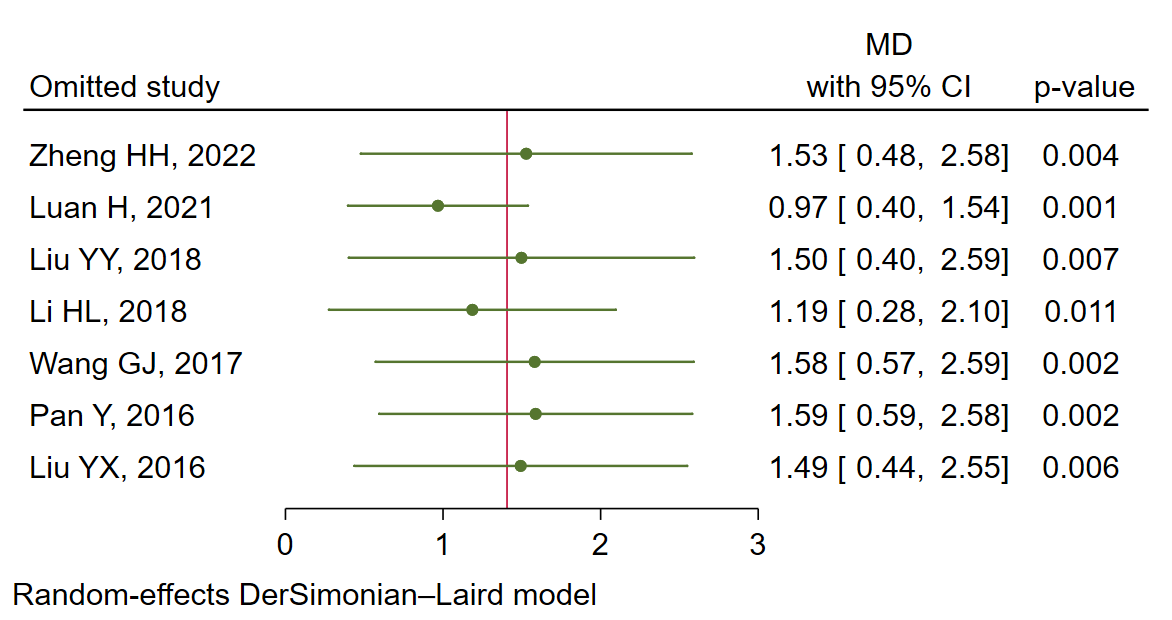


**Figure 3 Leave-one-out meta-analysis plot of total sleep time**


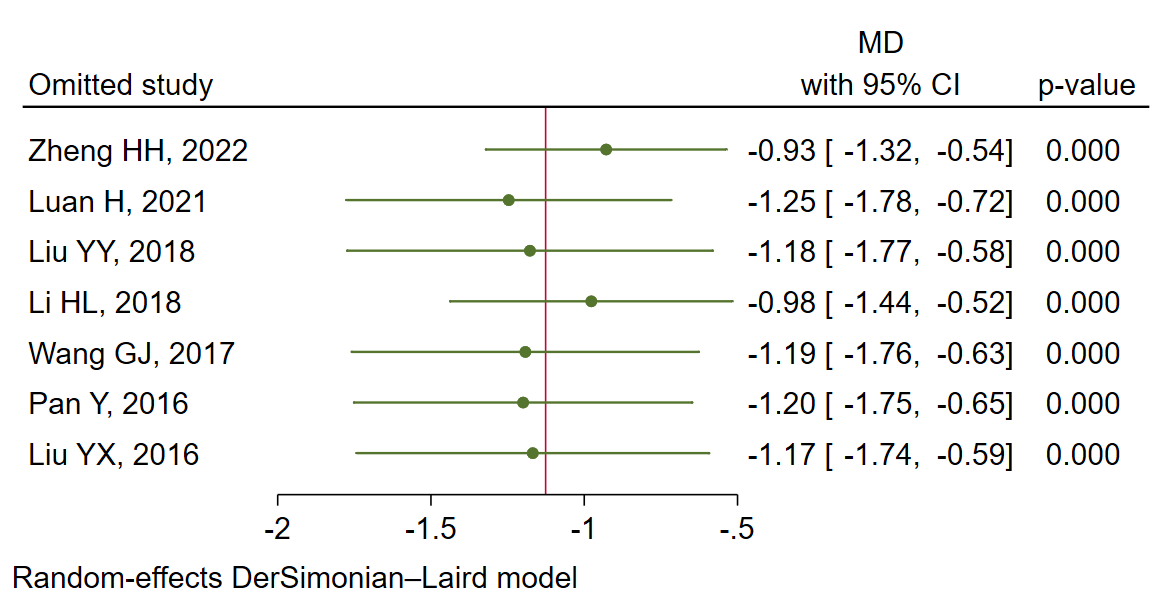


**Figure 4 Leave-one-out meta-analysis plot of awakening frequency**

**
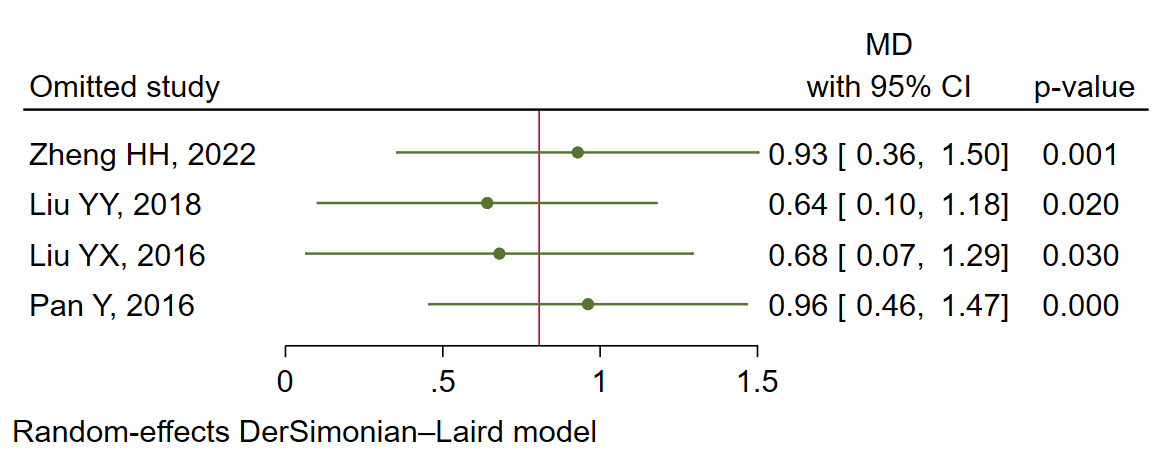
**

**Figure 5 Leave-one-out meta-analysis plot of REM time**

**
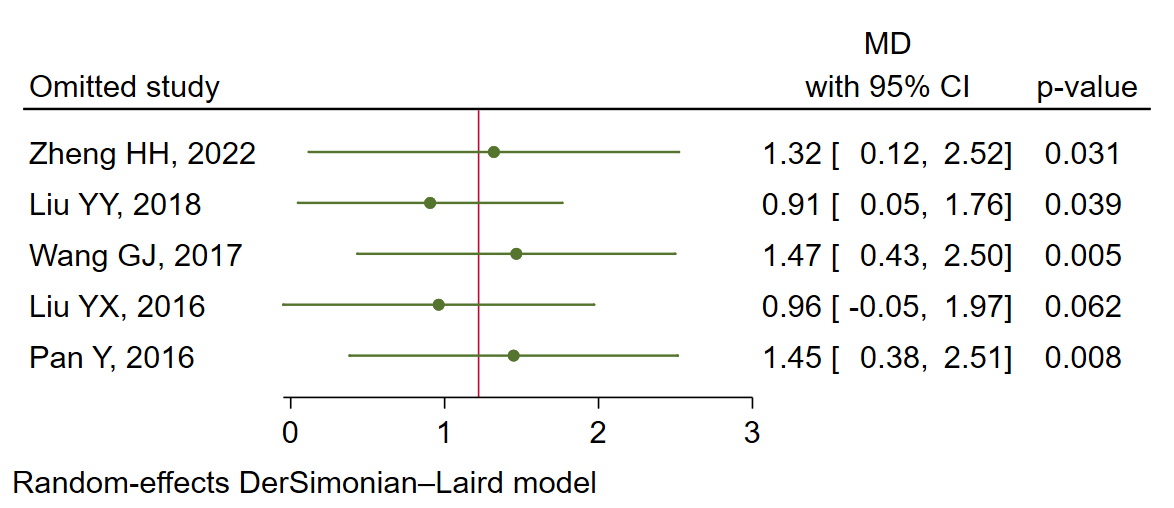
**

**Figure 6 Leave-one-out meta-analysis plot of SWS time**

**
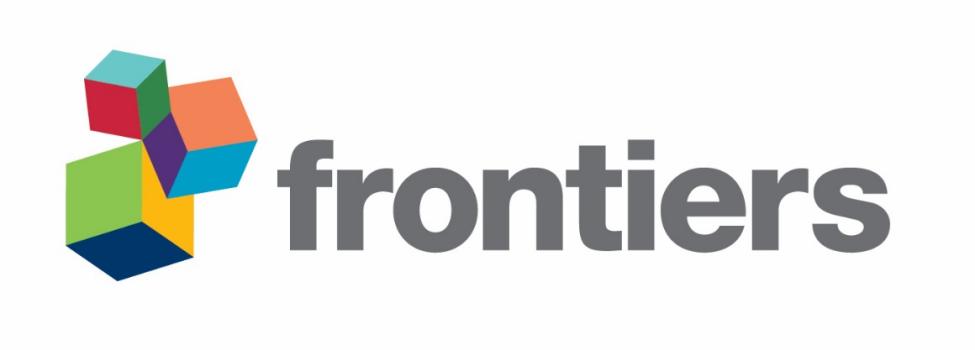
**
